# Unveiling Key Biomarkers of Cardiovascular Risk in Psoriasis Through Explainable Artificial Intelligence

**DOI:** 10.3390/biology15070532

**Published:** 2026-03-26

**Authors:** Hasan Ucuzal, Mehmet Kıvrak

**Affiliations:** 1 Department of Biostatistics, and Medical Informatics, Faculty of Medicine, Inonu University, Malatya 44280, Turkey; hasan.ucuzal@inonu.edu.tr; 2 Department of Biostatistics, and Medical Informatics, Faculty of Medicine, Recep Tayyip Erdogan University, Rize 53020, Turkey

**Keywords:** cardiovascular disease, psoriasis, machine learning, gradient boosting, explainable artificial intelligence, SHAP, biomarkers, class imbalance

## Abstract

Psoriasis patients face a significantly elevated risk of cardiovascular diseases (CVD), necessitating early and accurate risk prediction tools. This study developed and validated a machine learning model to predict CVD risk in psoriasis patients using clinical and biochemical data from 2685 individuals. After preprocessing and addressing class imbalance with SMOTE-NC, six machine learning models (Logistic Regression as baseline, XGBoost, LightGBM, CatBoost, GradientBoosting, AdaBoost) were evaluated using a completely leak-free nested cross-validation framework (outer k = 10, inner k = 3) with randomized hyperparameter search (n_iter = 50). Feature selection via the Boruta algorithm was performed separately within each training fold to prevent data leakage. The Boruta algorithm identified 21 key predictors, including age, systolic blood pressure (SBP), apolipoprotein B (apoB), fasting blood glucose (FBG), and complement C1q. CatBoost emerged as the top-performing model (OOF ROC-AUC = 0.908, 95% CI [0.892–0.924]; PR-AUC = 0.509, 95% CI [0.448–0.578]; F1 = 0.540; MCC = 0.498; Brier = 0.078), while the Logistic Regression baseline achieved ROC-AUC = 0.909 but was eliminated due to poor calibration (Brier = 0.114 > 0.10). All metrics were evaluated with 95% bootstrap confidence intervals (*n* = 1000 iterations). Explainable AI techniques (SHAP, LIME, Anchors) revealed that older age, elevated SBP, and metabolic dysregulation (e.g., high apoB, FBG) were the strongest CVD predictors. Local explanations were provided for five representative patients (high-risk, low-risk, and randomly selected), rather than a single instance, to better characterize model stability. Limitations include the single-center, retrospective design and lack of external validation. Future work should incorporate multi-ethnic cohorts and advanced biomarkers (e.g., genetic, imaging data) to enhance generalizability. This study demonstrates the potential of explainable AI to improve CVD risk stratification in psoriasis patients, offering a scalable tool for preventive cardiology.

## Simple Summary

Psoriasis patients face a substantially elevated cardiovascular disease (CVD) risk, yet early identification remains difficult with standard clinical tools. Using data from 2685 psoriasis patients, we trained and validated six machine learning models within a rigorous, leakage-free framework. CatBoost achieved the best performance (ROC-AUC = 0.908, Brier = 0.078) and identified 21 key predictors including age, blood pressure, and inflammatory markers. Explainable AI methods (SHAP, LIME, Anchors) provided transparent, patient-level risk explanations. This study demonstrates that interpretable machine learning can support CVD risk stratification in psoriasis patients using routine clinical data, pending external validation.

## 1. Introduction

Psoriasis is a chronic skin disorder characterized by a strong genetic predisposition and influenced by the immune system. Environmental factors can further exacerbate the condition. Its global prevalence is approximately 2%, though this varies regionally. It is more common in wealthier countries and in areas with a higher elderly population, as it tends to occur more frequently in adults than in children. The clinical presentation depends on the variant of psoriasis. Plaque psoriasis is the dominant form, affecting 80–90% of cases, and is characterized by well-demarcated erythematous, scaly patches or plaques [1]. Psoriasis is considered a systemic disease with multiple comorbidities, including depression, psoriatic arthritis, metabolic disorders such as obesity, diabetes, cardiovascular diseases (CVD), and metabolic syndrome [2]. Among these, CVD is one of the leading causes of death in patients with psoriasis. While psoriasis has been identified as a significant risk factor associated with CVD [3], severe forms of the disease are thought to independently predict cardiovascular risk [4]. Notably, CVD is among the leading causes of death and disability worldwide [5].

CVD is defined as a group of cardiac and vascular disorders, including ischemic stroke (IS), atrial fibrillation (AF), heart failure (HF), myocardial infarction (MI), and valvular heart disease (VHD), among others [6]. In 2020, approximately 19 million deaths worldwide were attributed to CVD. This estimate represents an 18.7% increase in the number of CVD-related deaths over the decade leading up to 2020 [7]. CVD is often the result of a combination of multiple etiologies [8]. The onset and progression of CVD may be driven by the interaction of genetic factors, environmental stimuli, and immune system dysfunctions [9]. Moreover, the symptoms of cardiovascular diseases are highly variable, and some individuals may present with atypical symptoms, which can lead to delays in diagnosis and treatment. Therefore, the development of a cardiovascular disease prediction model specifically for patients with psoriasis is of vital importance [10].

Nowadays, machine learning algorithms play an increasingly important role in supporting clinical decision-making across various medical applications [11]. With advances in data science, computing technologies, and machine learning is steadily gaining prominence in the healthcare field, where vast and diverse data types such as images, videos, and more are widely available [12]. These technological improvements aim to make healthcare services in certain domains more efficient, cost-effective, and dependable [13]. CVD continues to affect a large portion of the global population, leading to severe complications and even death. Many patients also face challenges accessing adequate healthcare due to limited time and overcrowded medical facilities. For these reasons, timely and precise identification of heart diseases is critical to improving patient outcomes. However, the complexity of the cardiovascular system, the delayed appearance of clinical symptoms, and individual genetic variability often hinder appropriate and timely treatment. To support healthcare professionals in overcoming these challenges, it is essential to develop reliable prediction systems for heart disease [14]. In order to facilitate rapid diagnosis and intervention of CVD in individuals with psoriasis and effectively delay disease progression, this study aimed to develop an interpretable CVD risk prediction model for psoriasis patients using multiple machine learning algorithms, evaluated within a leak-free nested cross-validation framework and explained through explainable AI techniques.

## 2. Materials and Methods

In this study, open-access data from 2685 psoriasis patients who presented to the Department of Dermatology at Beijing Traditional Chinese Medicine Hospital affiliated with Capital Medical University between 5 January 2009 and 1 September 2024 were utilized. During data collection, patients under 18 years of age (*n* = 32), those with malignant tumors (*n* = 31), and those with missing primary data (*n* = 7) were excluded. To ensure data consistency, only the first recorded visit was used for patients with multiple presentations [10].

### 2.1. Diagnostic Criteria

Psoriasis diagnosis was based on the criteria for psoriasis vulgaris (PV), pustular psoriasis (PP), erythrodermic psoriasis (EP), and psoriatic arthritis (PsA). Cardiovascular diseases (CVD) were diagnosed based on symptoms of acute coronary syndrome, myocardial infarction, stroke, or unstable angina, or a history of coronary artery bypass graft, percutaneous coronary intervention, or thrombolytic therapy. Dyslipidemia was diagnosed if at least one of the following criteria was met: total cholesterol (TC) ≥ 6.2 mmol/L, low-density lipoprotein cholesterol (LDL-C) ≥ 4.1 mmol/L, high-density lipoprotein cholesterol (HDL-C) < 1.0 mmol/L, triglycerides (TG) ≥ 2.3 mmol/L, or current use of lipid-lowering therapy. Hypertension was diagnosed based on office blood pressure (OBP) ≥ 140/90 mmHg (confirmed by ≥2 separate measurements), home blood pressure monitoring (HBPM) ≥ 135/85 mmHg (5–7 day average), or ambulatory blood pressure monitoring (ABPM) showing 24 h average ≥ 130/80 mmHg, daytime average ≥ 135/85 mmHg, or nighttime average ≥ 120/70 mmHg (including patients receiving antihypertensive treatment) [10].

### 2.2. Model Pipeline

**Figure 1:**
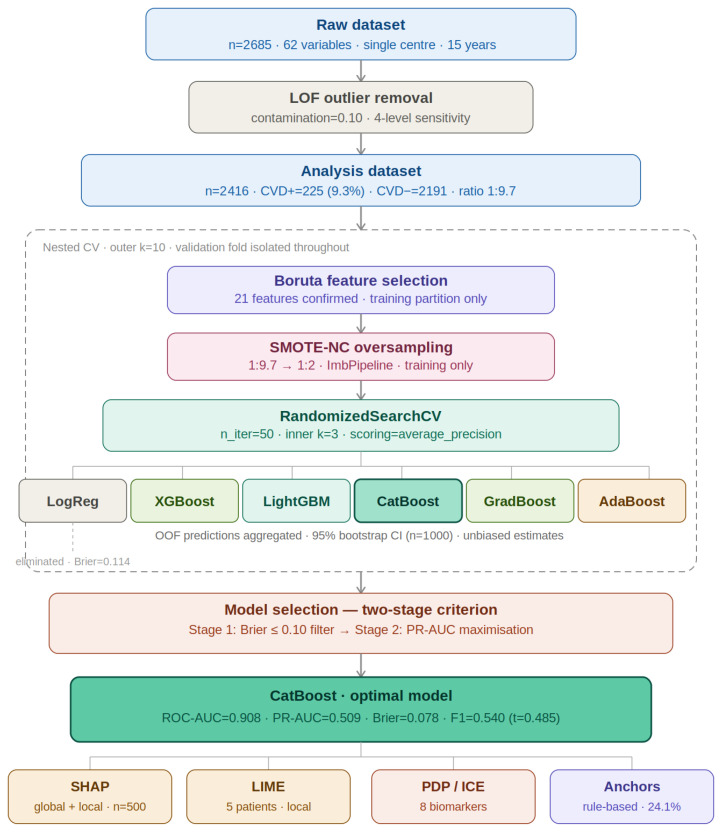
Workflow of the machine learning pipeline used in this study.

A structured machine learning pipeline was implemented to ensure strict separation between training and validation data and to prevent information leakage (Figure 1).

Dataset preparation: The initial dataset comprised 2685 individuals with 62 variables collected over a 15-year period from a single center. Potential outliers were identified using the Local Outlier Factor (LOF) algorithm with a contamination parameter of 0.10. After removal, the final analysis dataset included 2416 subjects, consisting of 225 CVD-positive cases (9.3%) and 2191 controls (class ratio ≈ 1:9.7).

Nested cross-validation framework: Model development was performed using nested cross-validation, with 10 outer folds for unbiased performance estimation and 3 inner folds for hyperparameter optimization. Within each outer fold, all preprocessing and modeling steps were applied exclusively to the training partition, while the validation fold remained completely isolated.

Feature selection and class imbalance handling: Feature selection was performed within each training partition using the Boruta algorithm, which identified 21 confirmed predictors. To address class imbalance, SMOTE-NC oversampling was applied only to the training data using the Imbalanced-Learn pipeline, improving the minority class representation.

Model training and optimization: Hyperparameters were optimized using RandomizedSearchCV (n_iter = 50) within the inner cross-validation loop. Optimization was based on average precision (PR-AUC), which is more informative for imbalanced classification tasks. Six algorithms were evaluated: Logistic Regression, XGBoost, LightGBM, CatBoost, Gradient Boosting, and AdaBoost. Performance estimates were derived from out-of-fold predictions, and 95% confidence intervals were obtained via bootstrap resampling (*n* = 1000).

Model selection: A two-stage selection strategy was applied. First, models with Brier score ≤ 0.10 were retained to ensure adequate probability calibration. Among these, the model with the highest PR-AUC was selected as the final model.

Final model and interpretability: Following this selection strategy, CatBoost achieved the best overall performance (ROC-AUC = 0.908, PR-AUC = 0.509, Brier = 0.078, F1 = 0.540). Model interpretability was assessed using SHAP, LIME, PDP/ICE, and Anchors to provide both global and local explanations.

### 2.3. Research Sample and Dataset

This case–control study aims to predict the prevalence of cardiovascular disease (CVD) in psoriasis using machine learning. The analysis included 2685 psoriasis patients (CVD+ = 259, CVD− = 2426) as the initial dataset, which was reduced to 2416 patients (CVD+ = 225, CVD− = 2191) following outlier removal. We employed comprehensive clinical and biochemical data to identify risk factors associated with CVD comorbidity. Predictors included demographic characteristics (age, sex), lifestyle factors (smoking, alcohol use), comorbidities (hypertension, diabetes, dyslipidemia), and inflammatory markers (neutrophil-to-lymphocyte ratio [NLR], systemic immune-inflammation index [SII], C-reactive protein [CRP]) (Table 1).

### 2.4. Data Preprocessing

The dataset was confirmed to have no missing values. Outlier detection was performed using the Local Outlier Factor (LOF) algorithm [15]. The parameters for LOF analysis were set with a number of neighbors of 20 and an expected outlier fraction of 0.10. Variables exhibiting high standard deviation, specifically SII, D-Dimer, LPa, UA, CK, LDH, and CRP, were included in the outlier analysis. As a result of this analysis, 269 outliers, constituting 10.02% of the dataset, were identified and subsequently removed, resulting in a final analysis dataset of 2416 patients (CVD+ = 225, CVD− = 2191). No data transformation methods were applied to the values within the dataset. To justify the contamination parameter (0.10), a sensitivity analysis was conducted by varying the LOF contamination rate across four levels: no LOF removal, 0.05, 0.10, and 0.15. The resulting CVD+ class proportions were 9.6% (*n* = 259), 9.3% (*n* = 237), 9.3% (*n* = 225), and 8.7% (*n* = 199), respectively, confirming that the class balance remained stable across all settings. This robustness analysis supports the choice of contamination = 0.10 as a clinically reasonable threshold for biochemical outlier detection in this population. The removed cases were not individually reviewed for clinical records as this is a secondary analysis of an anonymized public dataset.

### 2.5. Feature Selection

The Boruta algorithm [16] was employed for feature selection. To prevent data leakage, Boruta was executed independently within each outer cross-validation fold, applied solely to the training partition. A reference Boruta run on the full clean dataset confirmed a total of 21 predictive features, which were used for final model training and explainability analysis. The selected features are: C1q, P, LDH, apoB, apoE, GA, FBG, TP, ALB, PLT_PCT, GGT, Crea, Urea, NLR, PLT, Age, SBP, Hypertension, Diabetes, Dyslipidemia, and CK_MB.

### 2.6. Class Imbalance Handling

An analysis of the dataset confirmed the presence of class imbalance (CVD+:CVD− = 1:9.7). To address this, we applied the Synthetic Minority Over Sampling Technique for Nominal and Continuous features (SMOTE-NC) [17] exclusively within the training partition of each cross-validation fold to prevent data leakage, achieving a minority-to-majority ratio of approximately 1:2 after resampling. To assess whether SMOTE-NC introduced artificial patterns that could inflate performance, a sensitivity analysis was conducted by re-evaluating the best-performing model (CatBoost) without oversampling, with both conditions optimized via randomized grid search. The results showed ΔROC-AUC = +0.005, ΔPR-AUC = +0.019, and ΔBrier = +0.017, confirming that SMOTE-NC provided meaningful benefit for minority class detection without artificially inflating model performance.

### 2.7. Machine Learning Models and Hyperparameter Tuning

Six machine learning models were constructed for this study, including a Logistic Regression baseline and five boosting-based algorithms: GradientBoosting, CatBoost, AdaBoost, XGBoost, and LightGBM. Logistic Regression with L2 regularization served as the interpretable clinical baseline to contextualize the added predictive value of ensemble methods.

Hyperparameter optimization was performed using RandomizedSearchCV (n_iter = 50, inner k = 3, scoring = ‘average_precision’) within each outer cross-validation fold, applied exclusively to the training partition to prevent data leakage. The most frequently selected parameters across folds were as follows:

- Logistic Regression: Logistic Regression is a linear probabilistic classifier with L2 regularization, commonly used as a clinical baseline due to its interpretability and well-calibrated probability outputs [18]. The most frequently selected parameters were: C = 0.05, solver = saga, max_iter = 500, class_weight = balanced.
- GradientBoosting: GradientBoosting is a powerful ensemble learning technique that sequentially builds decision trees, with each new tree correcting errors made by the previous ones [19]. The most frequently selected parameters were: max_depth = 3, learning_rate = 0.05, n_estimators = 50, subsample = 0.5, min_samples_split = 30, min_samples_leaf = 10.
- CatBoost: CatBoost is an open-source gradient boosting library that automatically and effectively handles categorical features [20]. The most frequently selected parameters were: depth = 4, learning_rate = 0.03, iterations = 100, subsample = 0.6, l2_leaf_reg = 3, min_data_in_leaf = 30.
- AdaBoost: AdaBoost is an ensemble meta-algorithm that operates by weighting weak learners and iteratively improving their performance [21]. The most frequently selected parameters were: n_estimators = 200, learning_rate = 0.5.
- XGBoost: XGBoost is an optimized distributed gradient boosting library designed for efficiency, flexibility, and portability [22]. The most frequently selected parameters were: max_depth = 3, learning_rate = 0.05, n_estimators = 100, subsample = 0.8, reg_lambda = 1.0, reg_alpha = 1.0, min_child_weight = 3, colsample_bytree = 0.6.
- LightGBM: LightGBM is a gradient boosting framework that uses tree-based learning algorithms, known for its high speed and efficiency [23]. The most frequently selected parameters were: max_depth = 4, learning_rate = 0.03, n_estimators = 100, num_leaves = 20, subsample = 0.8, reg_lambda = 1.0, reg_alpha = 0.0, min_child_samples = 10.

### 2.8. Validation Strategy

A completely leak-free nested cross-validation framework was employed. The outer loop used Stratified K-Fold (k = 10) for unbiased out-of-fold (OOF) performance estimation. Within each outer fold, the following steps were applied exclusively to the training partition: (1) Boruta feature selection [16], (2) SMOTE-NC oversampling via ImbPipeline, and (3) RandomizedSearchCV hyperparameter optimization using an inner Stratified K-Fold (k = 3, n_iter = 50, scoring = ‘average_precision’). The validation fold remained entirely unseen during all preprocessing and optimization steps, ensuring pipeline-level isolation from both feature selection and oversampling. Model performance was evaluated using OOF predictions aggregated across all 10 outer folds. All reported metrics (ROC-AUC, PR-AUC, F1, MCC, Brier score) are accompanied by 95% bootstrap confidence intervals (1000 iterations, stratified resampling) to quantify estimation uncertainty.

### 2.9. Model Explainability

Post hoc interpretability of the models was conducted using several techniques:

- LIME (Local Interpretable Model-agnostic Explanations [24]: Local predictions were analyzed for five representative patients (one high-risk CVD+, one low-risk CVD−, and three randomly selected) to characterize model stability and inter-patient variability, rather than relying on a single instance.
- SHAP (SHapley Additive exPlanations) [25]: SHAP was used to determine global feature importance and provide local explanations. The global SHAP sample size was set to *n* = 500, selected via stratified sampling (preserving the CVD+/CVD− ratio) from the clean dataset (post-LOF, *n* = 2416) to ensure class-balanced representation. Local force plots were generated for the same five representative patients.
- Partial Dependence Plots (PDP) and Individual Conditional Expectation (ICE) [26]: These techniques were applied to examine the marginal effects of high-impact features, specifically FBG, apoB, apoE, SBP, Age, GGT, C1q, and NLR.
- Anchors [27]: Rule-based explanations were generated for five representative patients. For the high-risk patient (CVD+), the anchor rule identified was: Age ≤ 43.00 AND Dyslipidemia ≤ 0.00 (precision = 0.979, coverage = 0.241). The limited coverage (24.1%) reflects the local specificity of anchor rules in high-dimensional clinical data and should not be interpreted as a generalizable population-level rule.

## 3. Results

The study employed a systematic approach to address class imbalance and feature selection prior to modeling. Following LOF outlier removal, a class imbalance analysis confirmed a significant disparity in the target class distribution (CVD+ = 9.3%, CVD− = 90.7%). To mitigate this imbalance, SMOTE-NC was applied exclusively within each training fold.

Feature selection via the Boruta algorithm identified 21 confirmed predictors, including clinical and biochemical markers such as C1q, P, LDH, apoB, apoE, GA, FBG, TP, ALB, PLT_PCT, GGT, Crea, Urea, NLR, PLT, Age, SBP, and comorbidities Hypertension, Diabetes, and Dyslipidemia. Features not confirmed by Boruta (e.g., CRP, Neut, D-Dimer) were excluded.

For model development, six algorithms were evaluated: Logistic Regression (baseline), GradientBoosting, CatBoost, AdaBoost, XGBoost, and LightGBM. Hyperparameter optimization via RandomizedSearchCV (n_iter = 50, inner k = 3) identified optimal configurations for each model as detailed in Section 2.7. Model validation employed a completely leak-free nested cross-validation framework (outer k = 10, inner k = 3), ensuring that Boruta feature selection, SMOTE-NC oversampling, and hyperparameter optimization were applied exclusively within each training fold, providing unbiased OOF performance estimates. The Logistic Regression baseline achieved ROC-AUC = 0.909 (95% CI [0.890–0.925]) but was eliminated from best-model selection due to poor calibration (Brier = 0.114 > 0.10).

### 3.1. Correlation Analysis

**Figure 2:**
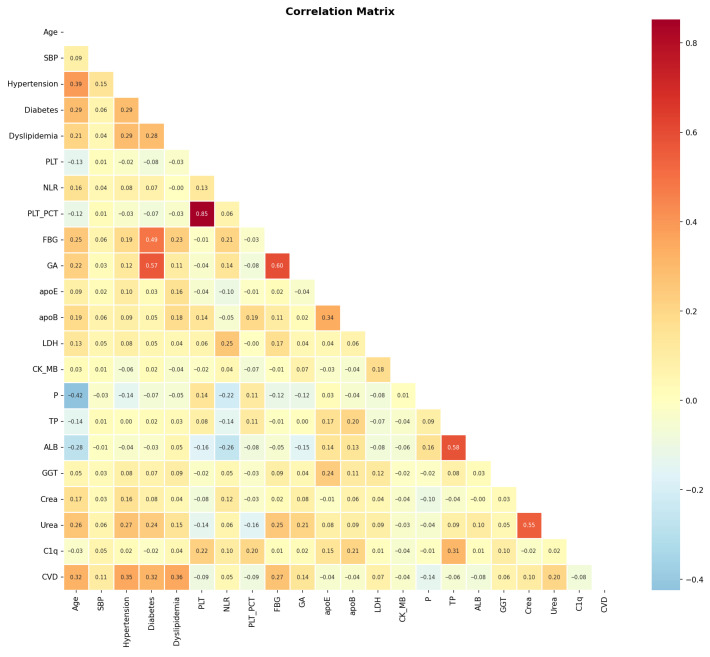
Correlation matrix of clinical and biochemical variables in the study dataset. Color intensity reflects the magnitude of Pearson correlation coefficients, with red indicating positive and blue indicating negative associations.

The correlation matrix (Figure 2) revealed associations between CVD and several clinical and biochemical markers. Among the strongest positive correlates of CVD, Dyslipidemia (r = 0.36), Hypertension (r = 0.35), Diabetes (r = 0.32), and Age (r = 0.32) were observed, consistent with their established roles as cardiovascular risk factors. FBG (r = 0.27) and Urea (r = 0.20) also demonstrated modest positive associations with CVD, suggesting contributions from glycemic dysregulation and renal function, respectively. P (r = −0.14), PLT_PCT (r = −0.09), and ALB (r = −0.08) exhibited weak negative correlations with CVD; these statistical associations warrant further investigation and should not be overinterpreted without experimental validation.

Complement C1q showed a weak negative correlation with CVD (r = −0.08). Given that Pearson correlation captures only linear relationships and does not account for confounding variables, the direction and magnitude of this association were further examined using SHAP global importance and partial dependence plots (see Section 3.3).

Among inter-variable correlations, PLT and PLT_PCT showed a strong positive correlation (r = 0.85), indicating collinearity between platelet-related indices. Diabetes demonstrated robust correlations with FBG (r = 0.49) and GA (r = 0.57), reflecting glycemic dysregulation. ALB showed a strong positive correlation with TP (r = 0.58), and Urea showed a strong positive correlation with Crea (r = 0.55), consistent with shared renal pathophysiology. LDH and CK_MB showed a modest positive correlation (r = 0.18), suggesting shared pathways of tissue injury. Hypertension correlated with Age (r = 0.39) and SBP (r = 0.15), reinforcing their interrelated roles in cardiovascular health.

### 3.2. Model Performance

The out-of-fold (OOF) performance of all six models is presented in Table 2 with 95% bootstrap confidence intervals. The Logistic Regression baseline achieved ROC-AUC = 0.909 (95% CI [0.890–0.925]), establishing a strong linear reference, but was eliminated from best-model consideration due to poor calibration (Brier = 0.114 > 0.10 threshold). Among the boosting models, CatBoost achieved the highest PR-AUC = 0.509 (95% CI [0.448–0.578]) and ROC-AUC = 0.908 (95% CI [0.892–0.924]), and was therefore selected as the optimal model. At t = 0.5: F1 = 0.534, MCC = 0.488, Brier = 0.078; at the optimal threshold (t = 0.485, MCC maximization): F1 = 0.540, Precision = 0.441, Recall = 0.698, MCC = 0.498. Notably, LightGBM demonstrated the best calibration among all models (Brier = 0.076), suggesting it may be preferable in settings where probability accuracy is the primary concern. The absolute discriminative gain of the best boosting model over the baseline was negligible (ΔROC = −0.0002), confirming that the primary advantage of ensemble methods lies in probability calibration (lower Brier scores) and minority class detection (PR-AUC). AdaBoost showed the weakest calibration (Brier = 0.145, Log-Loss = 0.470). A calibration curve confirmed the reliability of CatBoost predicted probabilities. The SMOTE sensitivity analysis (grid-search optimized in both conditions) showed that SMOTE-NC improved ROC-AUC by +0.005 and PR-AUC by +0.019, confirming that oversampling provided meaningful benefit for minority class detection without artificially inflating performance.

### 3.3. Explainability Analysis

**Figure 3:**
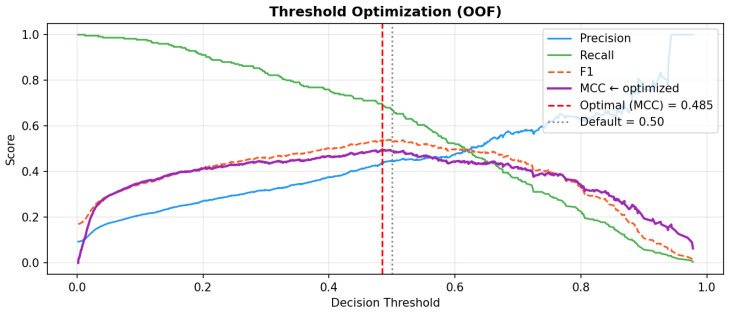
Decision threshold optimization for CatBoost based on out-of-fold predictions. Precision, Recall, F1-score, and MCC are plotted across the full threshold range [0, 1]. The optimal threshold (t = 0.485, red dashed line) was selected by maximizing MCC, yielding F1 = 0.540, MCC = 0.498, Precision = 0.441, and Recall = 0.698. The default threshold (t = 0.500, grey dotted line) is shown for comparison.

**Figure 4:**
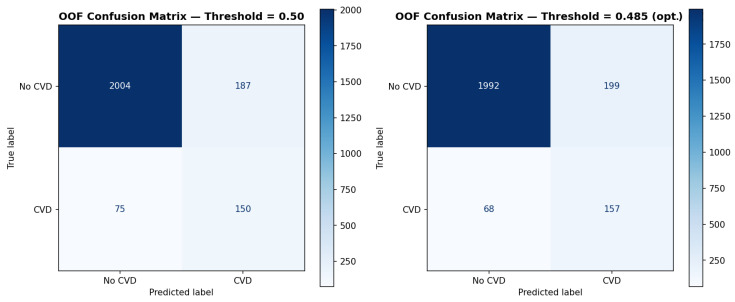
Out-of-fold confusion matrices for the CatBoost model at two decision thresholds. Left: default threshold (t = 0.500); Right: MCC-optimized threshold (t = 0.485). At t = 0.500: TP = 150, FP = 187, FN = 75, TN = 2004. At t = 0.485: TP = 157, FP = 199, FN = 68, TN = 1992. The optimized threshold improves minority class detection (Recall: 0.667 → 0.698) with a modest increase in false positives, reflecting the clinically appropriate trade-off for CVD screening in an imbalanced dataset.

The optimal decision threshold was determined by maximizing MCC across the OOF predictions, yielding t = 0.485 (Figure 3). At this threshold, CatBoost achieved F1 = 0.540, MCC = 0.498, Precision = 0.441, and Recall = 0.698. The corresponding OOF confusion matrix at both t = 0.500 and t = 0.485 is presented in Figure 4, demonstrating that the optimized threshold improved true positive detection (TP: 150 → 157) with a modest increase in false positives (FP: 187 → 199), reflecting the clinically appropriate trade-off between sensitivity and specificity in an imbalanced CVD screening context.

**Figure 5:**
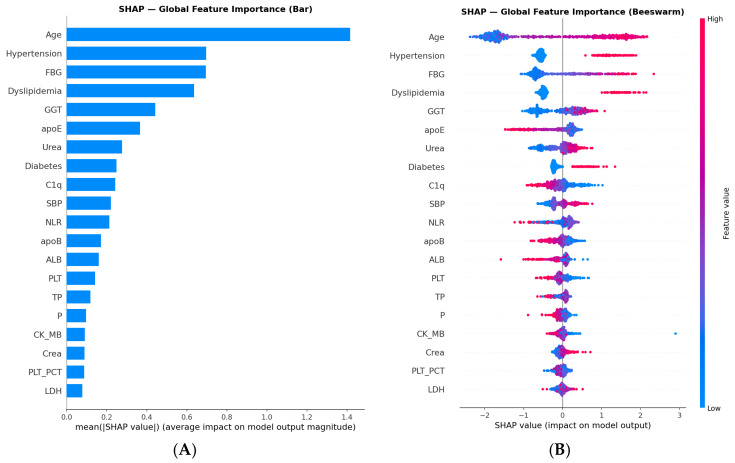

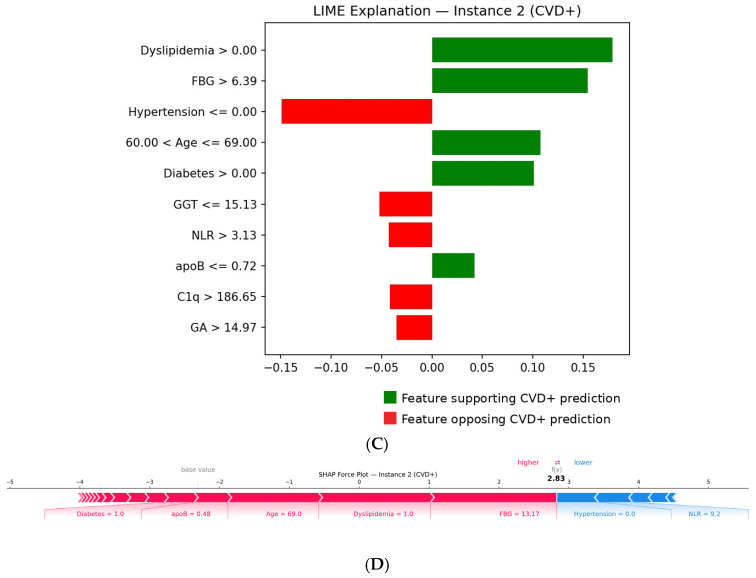
Explainability analysis of the CatBoost model. (**A**) SHAP global feature importance bar chart showing mean absolute SHAP values across all 21 selected features. (**B**) SHAP beeswarm plot illustrating the direction and magnitude of each feature’s contribution to model predictions (stratified sample, n = 500; color indicates feature value: red = high, blue = low). (**C**) LIME explanation for a representative high-risk patient (CVD+, Instance 2), detailing local feature contributions. (**D**) SHAP force plot for the same patient, visualizing individual feature impacts on the model output. LIME explanations and SHAP force plots for four other representative patients are provided in Supplementary Materials Figures S1–S4.

Global Feature Importance: SHAP analysis identified Age (mean |SHAP| ≈ 1.42), Hypertension (≈0.70), FBG (≈0.70), Dyslipidemia (≈0.65), and GGT (≈0.45) as the five most influential predictors of CVD risk in the CatBoost model (Figure 5). High Age values, presence of Hypertension and Dyslipidemia, elevated FBG, and elevated GGT were consistently associated with increased CVD risk (positive SHAP values). Notably, higher apoE (mean |SHAP| ≈ 0.38) and C1q (mean |SHAP| ≈ 0.23) values were associated with decreased CVD risk (negative SHAP values in beeswarm plot), consistent with their negative Pearson correlations with CVD (apoE: r = −0.04; C1q: r = −0.08). This inverse association of C1q with CVD risk was further confirmed by PDP analysis, which revealed a monotonic decrease in predicted CVD probability with increasing C1q levels, with the most pronounced inflection point around 130–140 mg/dL. Similarly, the PDP for apoE showed a decreasing trend in CVD probability with values above approximately 30–35 mg/dL. These findings suggest a potential protective role of complement C1q and apoE in this psoriasis cohort, though mechanistic interpretation requires experimental validation beyond the scope of this observational study.

Local Explanations (Five Representative Patients): LIME and SHAP analyses were performed on five patients (one confirmed CVD+, one confirmed CVD−, and three randomly selected) to characterize model behavior and inter-patient variability. For the high-risk patient (CVD+, Instance 2), the most influential LIME features were: Dyslipidemia > 0.00 (+0.203), FBG > 6.39 (+0.167), Hypertension ≤ 0.00 (−0.148), Diabetes > 0.00 (+0.106), and Age 60–69 (+0.103). The C1q feature showed a negative local contribution (−0.048), consistent with the global SHAP and PDP findings. Across the five patients, the relative importance rankings of the top features were consistent, supporting model stability. SHAP force plots for each patient are provided in the Supplementary Materials.

**Figure 6:**
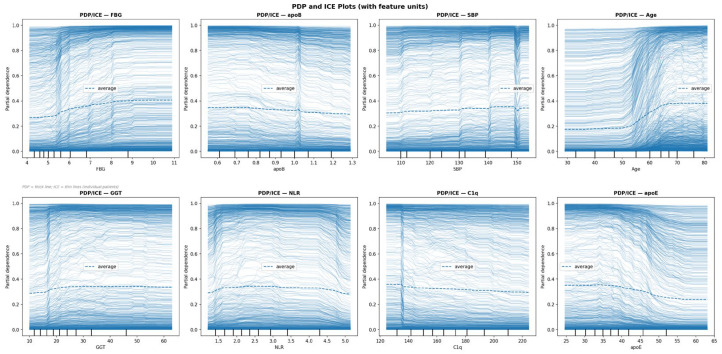
Partial Dependence Plots (PDP) and Individual Conditional Expectation (ICE) plots for eight key predictors selected by SHAP importance: FBG, apoB, SBP, Age, GGT, NLR, C1q, and apoE. Dashed lines represent the average partial dependence (PDP); thin lines represent individual patient trajectories (ICE). Monotonic increases in predicted CVD probability are observed with rising FBG (inflection ~6–7 mmol/L), Age (inflection ~55–60 years), and SBP (inflection ~150 mmHg). C1q and apoE demonstrate decreasing predicted CVD probability with higher values, consistent with SHAP global importance analysis. NLR shows an unexpected negative trend, warranting cautious interpretation.

PDP/ICE Plots: PDP and ICE plots revealed monotonic increases in predicted CVD probability with rising FBG (inflection ~6–7 mmol/L), Age (inflection ~55–60 years), and SBP (inflection ~150 mmHg). apoB showed a notably flat PDP curve, suggesting a weaker marginal effect than its SHAP ranking implies, likely reflecting interactions with other correlated features. NLR demonstrated an unexpected negative PDP trend; this counterintuitive finding may reflect population-specific confounding or feature interactions within the model and warrants cautious interpretation (Figure 6).

Anchors: For the high-risk CVD+ patient, the anchor rule identified was: Age ≤ 43.00 AND Dyslipidemia ≤ 0.00 (precision = 0.979, coverage = 0.241). The limited coverage (24.1%) reflects the local specificity of anchor rules in high-dimensional clinical data and should not be interpreted as a generalizable population-level rule.

**Figure 7:**
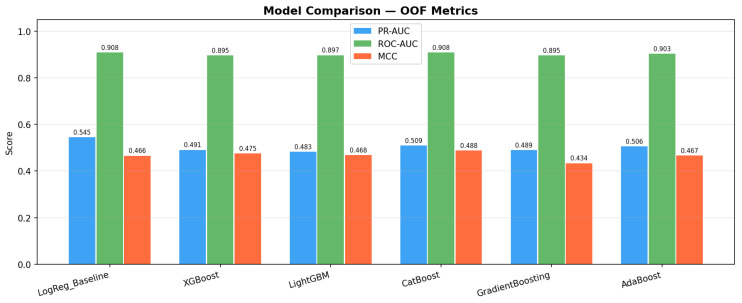
Model Performance Comparison (OOF Metrics).

The bar chart in Figure 7 compares the OOF performance of six models (including Logistic Regression baseline) across six evaluation metrics: ROC-AUC, PR-AUC, F1-Score, MCC, Brier Score, and Log-Loss, all reported with 95% bootstrap confidence intervals. CatBoost emerged as the top-performing model (ROC-AUC = 0.908, PR-AUC = 0.509), while the Logistic Regression baseline achieved ROC-AUC = 0.909 but was eliminated due to poor calibration (Brier = 0.114). The incremental discriminative gain of boosting models over the linear baseline is marginal (ΔROC ≈ −0.001), confirming that the predictive information in this dataset is largely capturable by linear models. However, boosting models showed substantially better probability calibration (Brier: 0.076–0.081 vs. 0.114 for LogReg), which is clinically important for risk stratification. LightGBM showed the best calibration (Brier = 0.076). AdaBoost showed the weakest calibration (Brier = 0.145, Log-Loss = 0.470).

**Figure 8:**
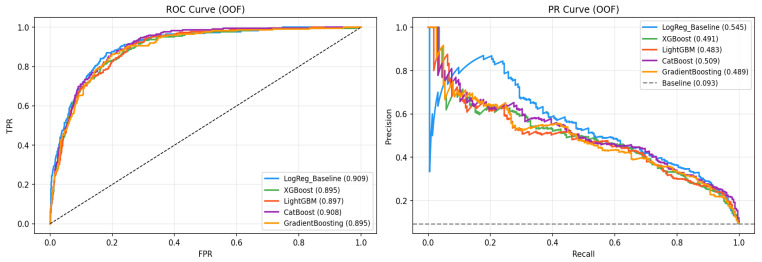
Out-of-fold ROC and Precision–Recall (PR) curves for all six models. The dashed diagonal line in the ROC plot represents the random classifier baseline. The horizontal dashed line in the PR plot represents the random baseline PR-AUC (≈0.096, equal to the positive class prevalence). CatBoost achieved the highest ROC-AUC = 0.908 (95% CI [0.892–0.924]) and PR-AUC = 0.509 (95% CI [0.448–0.578]) among calibration-eligible models. Logistic Regression is included as a linear baseline (ROC-AUC = 0.909) but was eliminated from best-model selection due to poor calibration (Brier = 0.114 > 0.10).

Figure 8 presents the OOF ROC and PR curves for all six models. CatBoost’s ROC curve achieved an AUC of 0.908 (95% CI [0.892–0.924]), reflecting strong discriminative power in this imbalanced dataset. The PR curve (AUC = 0.509, 95% CI [0.448–0.578]) reflects the genuine challenge of the 10:1 class imbalance and should be interpreted in the context of the random baseline PR-AUC (≈ 0.096), representing a 5.3× improvement. The Logistic Regression baseline ROC-AUC (0.909) is overlaid for comparison, confirming that the predictive information in this dataset is largely capturable by linear models, with CatBoost offering improved calibration (Brier = 0.078 vs. 0.114) and competitive minority class detection.

## 4. Discussion

The development and validation of a CVD prediction model for psoriasis patients represents a meaningful step in addressing cardiovascular risk in this population. Our study leveraged boosting algorithms and XAI within a leak-free nested cross-validation framework with randomized grid search (n_iter = 50, inner k = 3), yielding several key insights. CatBoost was identified as the optimal model by the study’s selection criteria (Brier ≤ 0.10 filter, then PR-AUC maximization): OOF ROC-AUC = 0.908, PR-AUC = 0.509, F1 = 0.540 (t = 0.485), MCC = 0.498, Brier = 0.078. Importantly, the Logistic Regression baseline achieved ROC-AUC = 0.909 but was eliminated due to poor calibration (Brier = 0.114 > 0.10). The absolute discriminative improvement of ensemble methods over the linear baseline is marginal (ΔROC ≈ −0.001). The primary advantages of CatBoost lie in its superior probability calibration (Brier = 0.078 vs. 0.114 for LogReg), better minority class detection (PR-AUC), and non-linear feature interaction modeling. Notably, LightGBM showed the best calibration among boosting models (Brier = 0.076), suggesting it may be preferable in settings where probability accuracy is paramount. The reported performance is substantially more conservative than previously reported values (AUC ≈ 0.98), which we attribute to the strict leak-free nested CV design with per-fold Boruta and pipeline SMOTE-NC eliminating preprocessing leakage.

The study’s results demonstrate that machine learning models, particularly CatBoost, can accurately predict CVD risk in psoriasis patients. Key predictors such as age, Hypertension, FBG, Dyslipidemia, and GGT were identified as the five most influential features by SHAP analysis, highlighting the interplay between metabolic dysfunction, chronic inflammation, and cardiovascular risk in this population. Notably, apoE (SHAP rank 6) and C1q (SHAP rank 9) demonstrated inverse associations with CVD risk, confirmed by both SHAP beeswarm and PDP analyses (Figure 4). Higher apoE values were associated with decreased predicted CVD probability (PDP inflection ~30–35 mg/dL), consistent with its established role in lipid clearance and anti-atherogenic function. C1q showed a similar inverse pattern (PDP inflection ~130–140 mg/dL), suggesting a potential protective role of complement activation in this psoriasis cohort. These associations are interpreted as statistical findings within this dataset and may reflect cohort-specific confounding rather than true protective mechanisms; mechanistic interpretation requires experimental validation beyond the scope of this observational study. These findings enable clinicians to identify high-risk patients using routine clinical and laboratory data. However, clinical translation requires prospective validation and the definition of actionable risk thresholds before implementation. The model’s strong probability calibration (Brier = 0.078) and its explainability (via SHAP/LIME/Anchors) represent prerequisites for real-world deployment, but integration into electronic health records or clinical workflows should be preceded by multi-center prospective testing [28,29].

Feature importance analysis confirmed that Age, Hypertension, FBG, Dyslipidemia, and GGT were dominant predictors, consistent with their established roles as cardiovascular risk factors. Aging exacerbates endothelial dysfunction and hypertension accelerates atherosclerosis in psoriasis patients. FBG highlights the significance of glycemic dysregulation, a component of metabolic syndrome prevalent in psoriasis. GGT, a marker of hepatic oxidative stress and metabolic dysregulation, has been increasingly recognized as an independent CVD risk factor [30]. ApoB (SHAP rank 12) showed a notably flat PDP curve despite its established role as an atherogenic lipid marker, suggesting its marginal contribution may be attenuated by interactions with correlated features in this cohort. NLR demonstrated an unexpected negative PDP trend; while NLR is generally regarded as an inflammatory marker associated with increased CVD risk, this counterintuitive finding may reflect population-specific confounding or feature interactions within the model and warrants cautious interpretation. Renal indices (Urea, Creatinine) and inflammatory markers reinforce the “psoriatic march” hypothesis, where chronic inflammation promotes end-organ damage [2]. The negative association of PLT_PCT with CVD (r = −0.09) is reported as a data-driven finding of modest magnitude requiring biological validation.

Our study addressed critical challenges in medical AI. Class imbalance (10:1) was mitigated via SMOTE-NC applied exclusively within training folds. A sensitivity analysis confirmed that SMOTE-NC improved ROC-AUC by +0.005 and PR-AUC by +0.019 compared to no oversampling (both conditions grid-search optimized), confirming that oversampling provided meaningful benefit for minority class detection without artificially inflating performance. Feature selection using Boruta eliminated redundant variables (e.g., CRP, D-Dimer), optimizing model parsimony. XAI techniques (SHAP, LIME, Anchors) provided clinician-friendly local and global interpretations [31]. We explicitly distinguish statistical feature importance from mechanistic causality and caution against over-interpreting these associations without supporting experimental evidence.

Despite robust performance, our study has several limitations. First, the single-center design using data from a Chinese tertiary hospital cohort [10] may limit generalizability to diverse populations, as tertiary hospitals tend to admit more severe cases, potentially overestimating comorbidity prevalence. Second, the retrospective design risks unmeasured confounders such as diet and genetic factors. Third, inflammatory markers such as CRP were excluded during feature selection, possibly undervaluing their role. Fourth, and most critically, external validation is absent. The nested CV framework provides unbiased internal estimates, but generalizability to other ethnic populations, healthcare settings, or time periods cannot be assumed. Prospective multi-center validation is required before clinical deployment.

Beyond immediate clinical utility, this study opens avenues for research into the mechanistic interplay between psoriasis and cardiovascular disease. The prominence of complement C1q and apoE suggests potential immunometabolic pathways that warrant further investigation. Future studies could integrate multi-omics data—such as genomics, proteomics, and metabolomics—to refine risk prediction and uncover subtype-specific CVD risks in psoriatic patients. Longitudinal monitoring using wearable devices could provide dynamic data inputs, enabling continuous risk stratification and personalized intervention strategies [32,33].

Based on these insights, future research directions should: (1) validate the model prospectively in multi-ethnic cohorts; (2) incorporate imaging data (e.g., coronary calcium scores) and genetic markers (e.g., HLA-C alleles) to enhance precision; and (3) test real-world implementation via electronic health record integration.

Our model offers tangible clinical benefits. High-risk patients (e.g., those with elevated FBG and Hypertension) could receive intensified preventive therapy or closer cardiovascular monitoring. Automating risk stratification may alleviate burdens in overcrowded dermatology settings [13]. The inverse association of C1q with CVD risk warrants further investigation into the potential role of complement pathways in psoriasis-associated cardiovascular disease.

## 5. Conclusions

This study developed a CVD prediction model for psoriasis patients using interpretable machine learning within a completely leak-free nested cross-validation framework with randomized grid search. CatBoost was identified as the optimal model (OOF ROC-AUC = 0.908, 95% CI [0.892–0.924]; PR-AUC = 0.509, 95% CI [0.448–0.578]; F1 = 0.540 (t = 0.485); MCC = 0.498; Brier = 0.078), with Logistic Regression serving as baseline (ROC-AUC = 0.909) but eliminated due to poor calibration (Brier = 0.114 > 0.10). LightGBM showed the best calibration among boosting models (Brier = 0.076). The marginal discriminative advantage of ensemble methods over the linear baseline was offset by substantially better probability calibration and minority class precision, which are clinically important for CVD risk stratification. By integrating demographic, metabolic, and inflammatory data with rigorous methodology (per-fold Boruta, pipeline SMOTE-NC, LOF sensitivity analysis), we identified key drivers of cardiovascular risk. XAI techniques (SHAP, LIME, PDP/ICE, Anchors) provided both global and patient-level interpretations across five representative patients. Future work must prioritize external validation in multi-ethnic cohorts and prospective intervention trials before clinical deployment. Comparisons with established risk scores (e.g., Framingham, ASCVD) should be pursued in subsequent studies.

## Figures and Tables

**Table 1:** Variables and Categories Table.

Category	Variables
Demographic Characteristics	Age, Gender, Admission Time
Medical History	Smoking, Alcohol Consumption, Hypertension, Diabetes, Dyslipidemia, Non-Metabolic Fatty Liver Disease, Malignancy, CVDs (Cardiovascular Diseases)
Clinical Characteristics	Systolic Blood Pressure, Diastolic Blood Pressure
Laboratory Tests	Hematology: WBC, PLT, Hb, RBC, Mono, Neut, Lymph, NLR, SII, MCV, RDW-CV, Hct, PDW, PLT_PCT, MPV, MCHC
	Biochemistry: FBG, apoE, apoB, apoAI, LPa, LDH, α-HBDH, CK, CK-MB, Ca, P, K, TP, PA, ALB, GLO, GGT, TBIL, DBIL, TBA, UA, Crea, Urea, AST, ALT
	Coagulation: PT, FDP, APTT, TT, D-Dimer
	Inflammatory Markers: CRP, ESR, C3, C4, IgA, IgG, C1q
	Other Indices: MHR, TyG

**Table 2:** Out-of-fold (OOF) model performance with 95% bootstrap confidence intervals (*n* = 1000 iterations). Models ranked by PR-AUC among calibration-eligible models.

Model	ROC-AUC (95% CI)	PR-AUC (95% CI)	F1 (t = opt)	MCC	Brier	Log-Loss
CatBoost *	0.908 [0.892–0.924]	0.509 [0.448–0.578]	0.540	0.498	0.078	0.252
XGBoost	0.895 [0.876–0.913]	0.491 [0.426–0.557]	0.523	0.476	0.079	0.253
GradientBoosting	0.895 [0.875–0.914]	0.490 [0.428–0.556]	0.489	0.434	0.081	0.275
LightGBM +	0.897 [0.879–0.914]	0.483 [0.421–0.555]	0.519	0.468	0.076	0.242
LogReg ~	0.909 [0.890–0.925]	0.545 [0.479–0.615]	0.489	0.466	0.114	0.376
AdaBoost ~	0.903 [0.886–0.919]	0.506 [0.442–0.572]	0.515	0.467	0.145	0.470

* Selected model (Brier ≤ 0.10 filter, then PR-AUC maximization). + Eliminated: Brier > 0.10. ~ Best calibration (Brier = 0.076). F1 reported at optimal threshold (CatBoost: t = 0.485; others: t = 0.500). Random baseline PR-AUC ≈ 0.096.

## Data Availability

The raw data supporting the conclusions of this article will be made available by the authors on request.

## Supplementary Materials

The following supporting information can be downloaded at: https://www.mdpi.com/article/10.3390/biology15070532/s1, Figure S1: LIME Explanation instance 0 (CVD-); Figure S2: LIME Explanation instance 410 (CVD-); Figure S3: LIME Explanation instance 199 (CVD-); Figure S4: LIME Explanation instance 1670 (CVD-).
