# Supplementary figures and images for: Unveiling Key Biomarkers of Cardiovascular Risk in Psoriasis Through Explainable Artificial Intelligence
Source: Biology (Basel). 2026 Mar 26;15(7):532. doi: 10.3390/biology15070532 (PMC13071970; doi:10.3390/biology15070532)

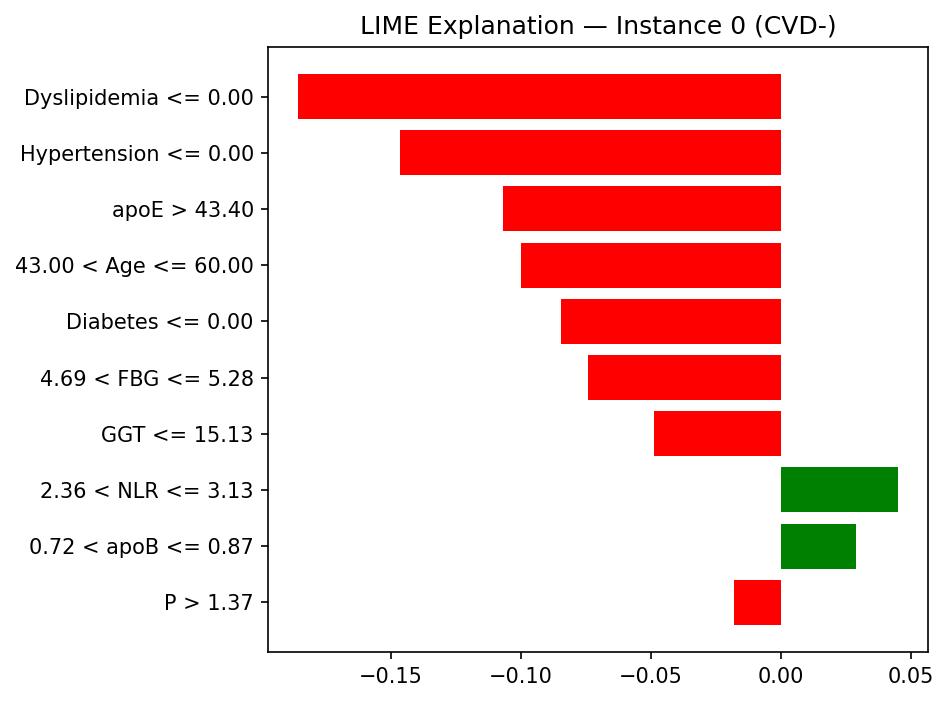

Supplement: Supplementary file 1 [file biology-15-00532-s001.zip › lime_explanation_inst2.png]

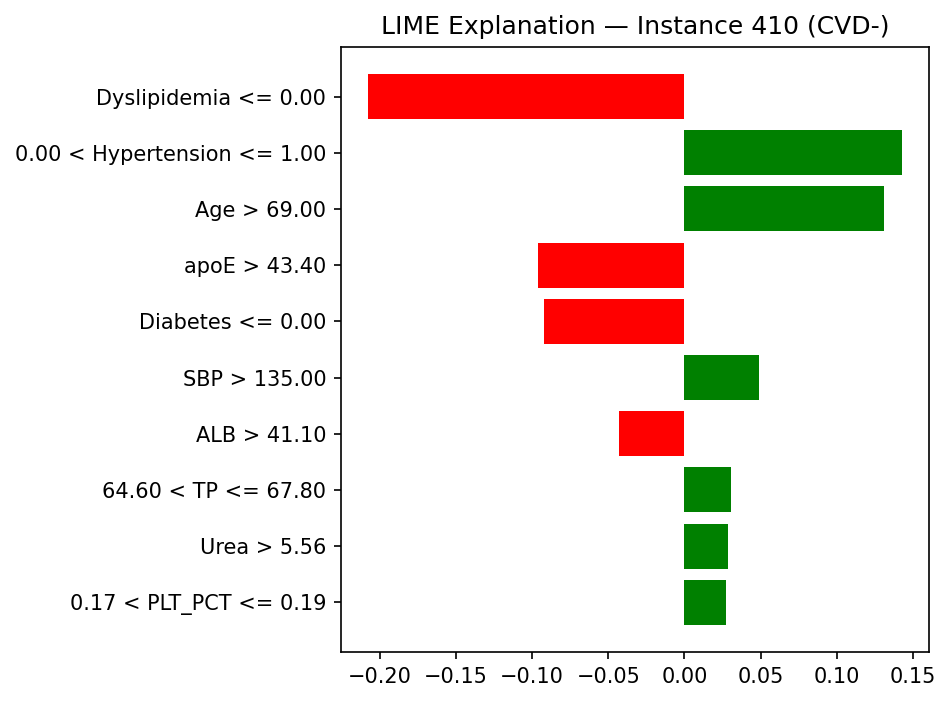

Supplement: Supplementary file 1 [file biology-15-00532-s001.zip › lime_explanation_inst3.png]

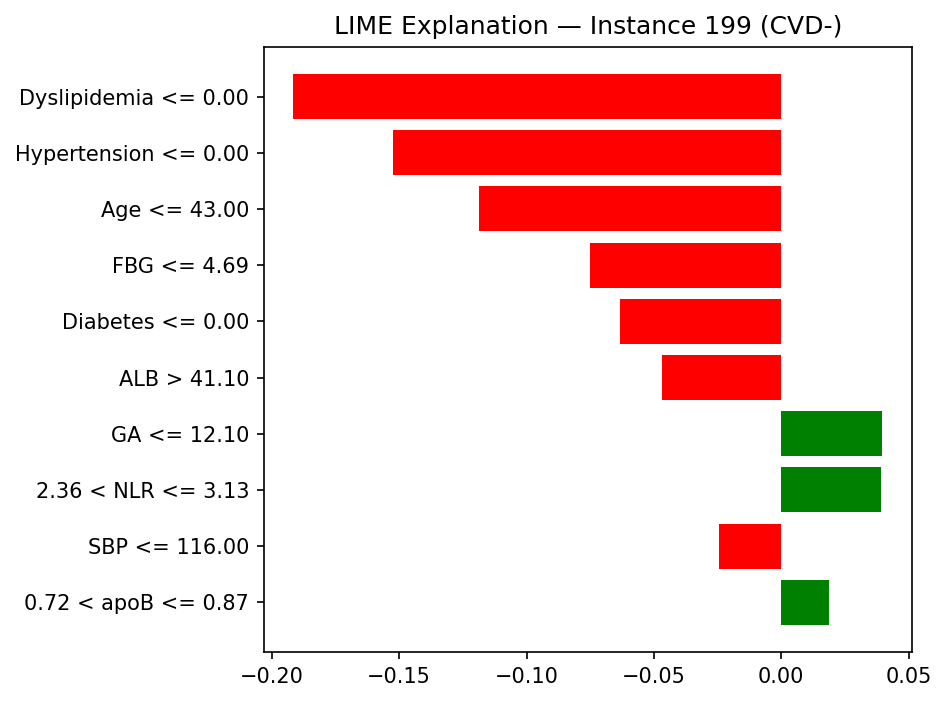

Supplement: Supplementary file 1 [file biology-15-00532-s001.zip › lime_explanation_inst4.png]

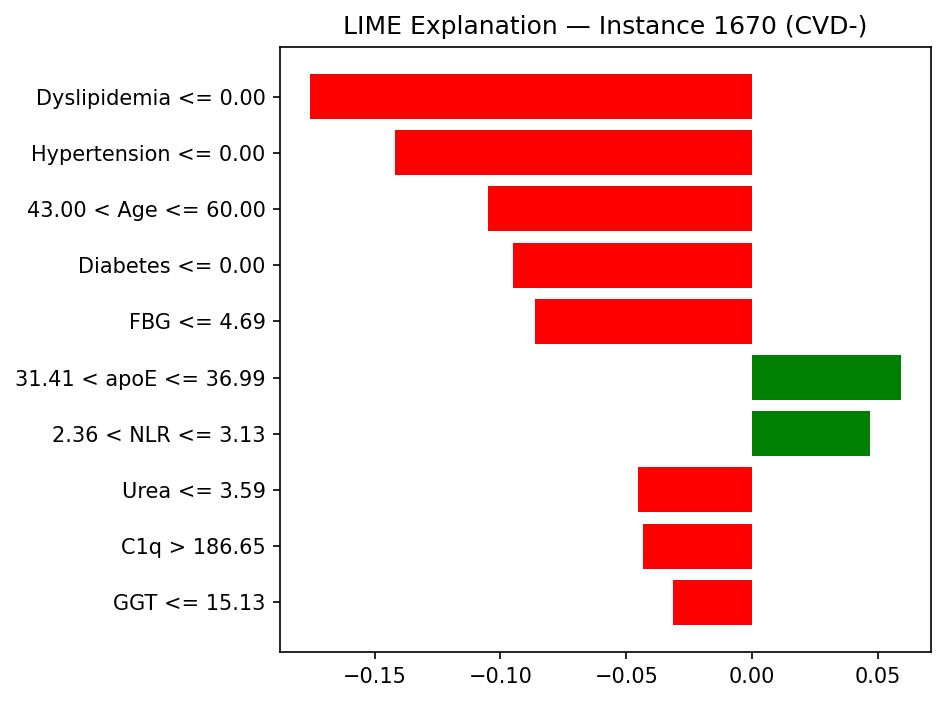

Supplement: Supplementary file 1 [file biology-15-00532-s001.zip › lime_explanation_inst5.png]
